# miRNALoc: predicting miRNA subcellular localizations based on principal component scores of physico-chemical properties and pseudo compositions of di-nucleotides

**DOI:** 10.1038/s41598-020-71381-4

**Published:** 2020-09-03

**Authors:** Prabina Kumar Meher, Subhrajit Satpathy, Atmakuri Ramakrishna Rao

**Affiliations:** ICAR-Indian Agricultural Statistics Research Institute, New Delhi, 12 India

**Keywords:** Computational biology and bioinformatics, Molecular biology

## Abstract

MicroRNAs (miRNAs) are one kind of non-coding RNA, play vital role in regulating several physiological and developmental processes. Subcellular localization of miRNAs and their abundance in the native cell are central for maintaining physiological homeostasis. Besides, RNA silencing activity of miRNAs is also influenced by their localization and stability. Thus, development of computational method for subcellular localization prediction of miRNAs is desired. In this work, we have proposed a computational method for predicting subcellular localizations of miRNAs based on principal component scores of thermodynamic, structural properties and pseudo compositions of di-nucleotides. Prediction accuracy was analyzed following fivefold cross validation, where ~ 63–71% of AUC-ROC and ~ 69–76% of AUC-PR were observed. While evaluated with independent test set, > 50% localizations were found to be correctly predicted. Besides, the developed computational model achieved higher accuracy than the existing methods. A user-friendly prediction server “miRNALoc” is freely accessible at https://cabgrid.res.in:8080/mirnaloc/, by which the user can predict localizations of miRNAs.

## Introduction

It has been established that the non-coding RNAs (ncRNAs) are important regulator rather than the junk sequences^1^. For variety of diseases, these are verified to be important biomarkers^2^. MicroRNAs (miRNAs) are one type of ncRNA^3^ that are ~ 20–22 nucleotides long^4^, contribute to a variety of cellular processes through their involvement in the regulation of gene expression^5–7^. In association with the Argonaute (AGO) proteins, miRNAs form the core component of the miRISC (miRNA-induced silencing complex) that regulates a wide range of intracellular processes. Although miRNAs are known to function as a component of RISC in the cytoplasm^8^, they have also been discovered in other cellular compartments including nucleus^9–11^, nucleolus^12^, mitochondria^11,13^, exosome^14,15^, extracellular vesicle^16^ and circulation^17–20^. As reported by Leung^21^, subcellular localization of miRNAs is critical to its function, particularly the discoveries of miRNAs in the nucleus^22^ and their ability to guide RNA target cleavage^23^. Besides, information on subcellular localizations would help in designing and interpreting miRNA profiling experiments, distortions of which are reported to be associated with various diseases including cancer^24–26^.

From the above studies, the importance of subcellular localization of miRNAs can be deduced. Although the biological experiments such as immunofluorescence confocal microscopy, subcellular fractionation and immunoprecipitation are reliable in locating the subcellular localizations, they are resource intensive. Computational methods can be good alternative to supplement the biochemical experiments. However, this has been done mostly for protein subcellular localization prediction^27–30^. In addition, few attempts have also been made towards RNA molecules. Specifically, Feng et al.^31^ established a computational approach for prediction of organelle location of ncRNAs. Further, Cao et al.^32^ established a computational tool “lncLocator” to predict the subcellular localizations of lncRNAs (long non-coding RNAs). In another study, iLoc-lncRNA was developed by Su et al.^33^ for subcellular localization prediction of lncRNAs. As far as predicting subcellular localization of miRNAs is concerned, only two approaches i.e., MiRGOFS-based predictor^34^ and miRLocator^35^ are available in literature, to the best of our knowledge. Though these approaches have achieved an acceptable level of accuracy, still there is room for improvement. Further, no computational tools or prediction servers are available for both the existing approaches. Thus, an attempt has been made in this study to establish an alternative computational method along with a computational tool for predicting multiple subcellular localizations of miRNAs. The pseudo-dinucleotide compositions along with the physico-chemical and thermodynamic properties of miRNAs were utilized as features, where the support vector machine (SVM)^36^ along with other machine learning methods were employed as predictor. The developed computational tool or prediction server is believed to supplement the research related to RNA biology.

## Methods

### Collection and processing of dataset

Construction of benchmark datasets is essential to develop any machine learning-based predictor. We downloaded the sub cellular localization details of miRNA sequences from RNALocate database^37^ available at https://www.rna-society.org/rnalocate/. A total of 9,456 miRNA sequences with curated subcellular locations information were retrieved. After removing redundancy, a total of 2,525 unique miRNA sequences were retained. Further exclusion of hairpin miRNA sequences resulted in 2,202 unique mature miRNA sequences distributed over 16 subcellular localizations (Supplementary Table S1). Out of 2,202, 1,292 were confined to unique (single) localization only while 910 were found to be present in more than one localization. After analyzing the sequences confined to single location, < 10 sequences were found for cell body, chloroplast, dendrite, endoplasmic reticulum, nucleolus, nucleoplasm, ribosome and synapse. Hence, we considered the miRNA sequences belonging to the remaining 8 subcellular localizations, where 1,270 were found to be present in single location and 691 sequences in more than one location.

### Positive and negative datasets

For each subcellular localization, both positive and negative datasets were prepared. For a given localization, the positive set constitutes the sequences belonging to that localization only and the negative set constitutes the remaining unique localization sequences (called as ND-I). We used another negative dataset (i.e., ND-II) that contains randomly drawn 1,000 miRNA sequences from miRBase database (https://www.mirbase.org/) whose localizations are not known. Hence, we assumed here that the sequences are from other localizations than the considered eight localizations. Further, to avoid homologous bias, 80% identical sequences were removed from both the positive and negative sets using CDHIT program^38^ with sequence identity cut-off 0.8. The 80% cutoff was employed based on the earlier studies involving nucleotide sequence data. Besides, employing a more stringent cutoff will further reduce the size of the dataset. Positive and negative datasets for each of the 8 localizations are summarized in Table 1.

**Table 1:** Summary of the positive and negative datasets.

Localization type	Positive	ND-I	ND-II
Axon	16	830	951
Circulating	69	775	
Cytoplasm	67	808	
Exosome	524	415	
Extracellular vesicle	25	829	
Microvesicle	21	818	
Mitochondrion	191	659	
Nucleus	42	799	

Last column represents the negative dataset collected from miRBase database. Number of sequences presented are obtained after removing redundancy with sequence identity cut-off 0.8 using CD-HIT program.

### Independent test dataset

**Figure 1:**
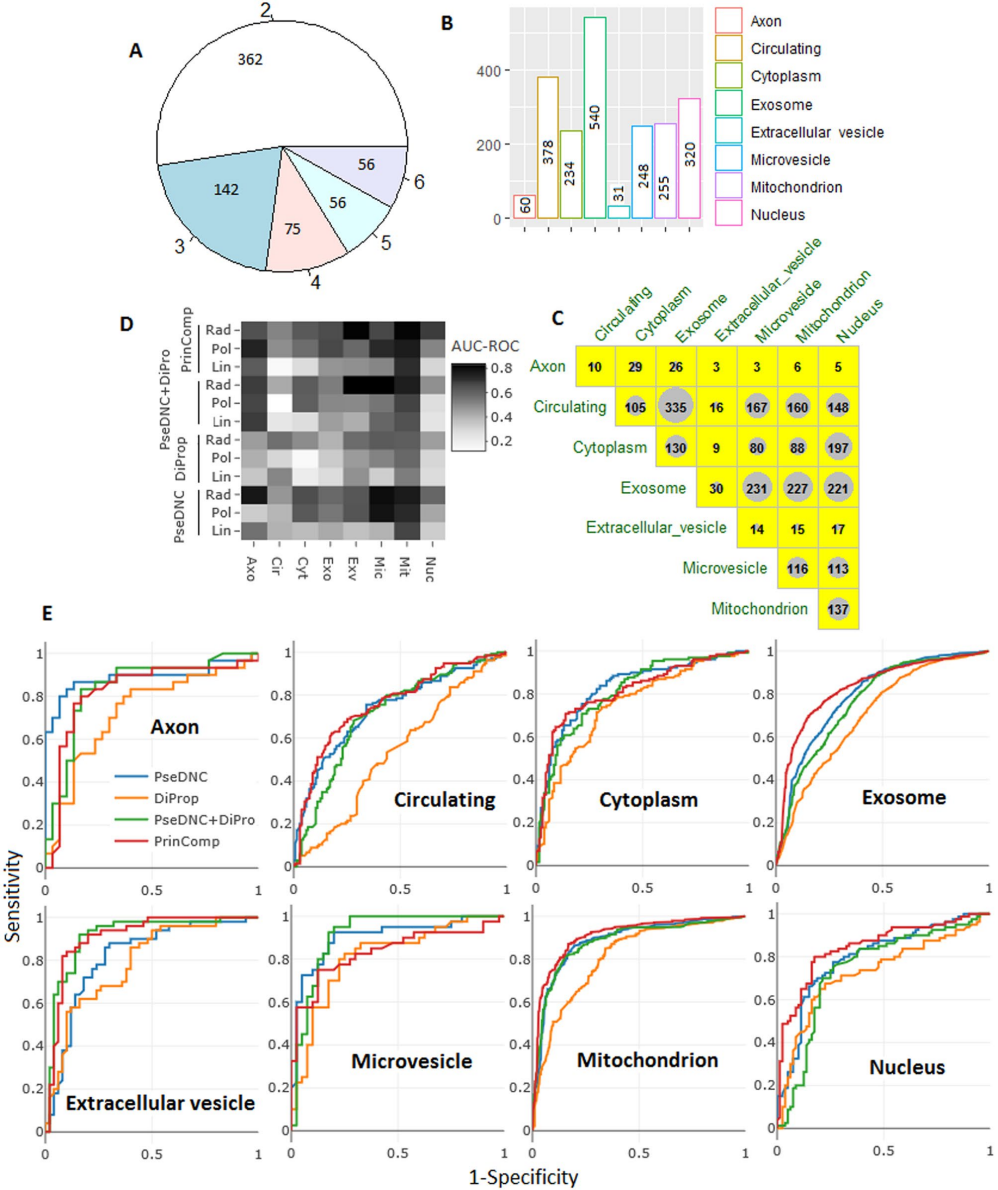
(**A**) Distribution of sequences of the test set over number of localizations. (**B**) Number of sequences of the test set present in different locations. (**C**) Distribution of sequences in more than one locations. (**D**) Heat map of AUC-ROC for four different kernels with all the four feature sets. (**E**) ROC curves for all the four festure sets with all the eight localizations.

The independent dataset was built with 691 miRNA sequences, where each sequence belonged to more than one localization. In other words, accuracy was evaluated with regard to the prediction of more than one subcellular localization of miRNAs. Among 691 sequences, more than 50% were present in 2 localizations, where the sequences were seen to be present in a maximum of six localizations (Fig. 1A). Less number of sequences were observed for axon and extracellular vesicle, whereas a large number of sequences (> 200) for other six localizations (Fig. 1B). Among all the localizations, larger number of sequences was found for the exosome. Further, most of the sequences present in other locations were also seen to be present in the exosome (Fig. 1C).

### Feature generation

Generation of discriminative features is crucial for achieving higher prediction accuracy in machine learning algorithm (MLA)-based prediction. Since miRNA sequences are shorter in size (20–22 nucleotides), generation of discriminative sequence-based features is challenging. Here, we utilized two types of features i.e., pseudo dinucleotide compositions (PseDNC)^39^ and di-nucleotide properties (DiPro) for RNA category obtained from DiProDB^40^ database. Besides, we also employed two different combinations of features i.e., PseDNC + DiPro and principal component scores of PseDNC + DiPro (we call it PrinComp). The Pse-in-One server^41^ was implemented for retrieving PseDNC features. Here, the purpose of using PrinComp feature is to transform the correlated features into independent features or predictors, rather than reducing the dimension. Therefore, all the principal component scores were subjected for prediction. The PrinComp features were nothing but the principal component scores obtained from the combined features of DiPro and PseDNC, where the function *princomp* of the R-package “stats”^42^ was utilized to get the principal component scores. A precise description of computation of features is as follows.

#### Features based on di-nucleotide properties (DiPro)

We extracted the physico-chemical, thermodynamic and conformational properties of di-nucleotides from DiProDB, which is accessible at https://diprodb.fli-leibniz.de/ShowTable.php. Specifically, 11 different properties of RNA i.e., twist, rise, shift, tilt, slide, roll, stacking energy, hydrophilicity, enthalpy, entropy and free energy are available in this database. However, there are two types of hydrophilicity, enthalpy, entropy and free energy. Thus, a total of 15 features were employed. The values of these properties corresponding to each di-nucleotide are given in Supplementary Table S2. Based on these properties, each sequence was mapped to a vector of 15 numeric observations, where each element corresponds to the mean value of the respective di-nucleotide properties.

#### Feature based on pseudo di-nucleotide composition (PseDNC)

With the tendency to capture both local and global ordering information of di-nucleotides^43,44^, PseDNC feature descriptor has been employed for sequence encoding in many fields of computational biology and bioinformatics^45–48^. For a given nucleotide sequence, the PseDNC feature vector can be represented as $V=\{{v}_{1}{v}_{2}\dots {v}_{16}{v}_{16+1}\dots {v}_{16+\lambda }\}$ with

$$v_{\tau } = \left\{ {\begin{array}{ll} {\frac{{g_{\tau } }}{{\sum _{{i = 1}}^{{16}} g_{i} + \omega \sum\limits_{{j = 1}}^{\lambda } {\alpha _{j} } }}} & {(1 \le \tau \le 16)} \\ {\frac{{w\alpha _{{\tau - 16}} }}{{\sum _{{i = 1}}^{{16}} g_{i} + \omega \sum\limits_{{j = 1}}^{\lambda } {\alpha _{j} } }}} & {(16 \le \tau \le 16 + \lambda )} \\ \end{array} } \right.,$$

where $w$ is the weight factor, $\lambda$ represents the number of pseudo components, ${\alpha }_{j}$ is the *j*th tier correlation factor and ${g}_{\tau }$ represents the normalized frequencies of di-nucleotides. The *j*th tier correlation factor is nothing but the correlation between all the *j*th adjacent di-nucleotides, and for any sequence of *L* nucleotides long it can be computed as ${\alpha }_{j}=\frac{1}{L-j-1} \sum_{i=1}^{L-j-1}{R}_{i,j} (j=\mathrm{1,2},\dots ,\lambda ;\lambda <L)$, where ${R}_{i,j}=\frac{1}{\mu }\sum_{f=1}^{\mu }[{P}_{f}\left({D}_{i}\right)-{{P}_{f}({D}_{j})]}^{2}$. Here, *µ* denotes the number of di-nucleotide properties which is 15 in this study, ${P}_{f}\left({D}_{i}\right)$ and ${P}_{f}\left({D}_{j}\right)$ are the numeric values of the ${f}^{th}$ di-nucleotide properties for the di-nucleotide at *ith* and *jth* positions of the sequence respectively.

### Prediction with SVM

The SVM has been effectively and successfully employed in several areas of bioinformatics^49–53^. A precise description about SVM can be found in Chou and Cai^54^. Based on structural risk minimization principle, SVM has strong generalization ability. The SVM algorithm searches for a hyper plane that maximizes the margin between observations of different classes. In this regard, the kernel function plays a crucial role^55^. We first assessed the accuracy with four widely used kernels (radial, sigmoid, polynomial and linear) using a sample dataset from each localization, and the kernel function that provided highest accuracy was utilized in the final prediction. The SVM was implemented with “e1071”^56^ package of R-software.

### Measuring prediction accuracy

Two important measures that are area under ROC (receiver operating characteristics) curve (AUC-ROC)^57^ and PR (precision-recall) curve (AUC-PR)^58^ are employed to assess the accuracy of prediction model. Besides, sensitivity = $tp/(tp+fn)$, specificity = $tn/(tn+fp)$, F1-score=$2\times precision\times recall/(precision+recall)$ and MCC=$=[(tp\times tn)-(fp\times fn)]/sqrt[(tp+fn)\times (tp+fp)\times (tn+fp)\times (tn+fn)]$ were also utilized to measure the prediction accuracy, where recall is same as the sensitivity for binary classification and precision = $tp/(tp+fp)$. The *tp, tn, fp* and *fn* denote true positive, true negative, false positive and false negative respectively. Further, repeated fivefold cross validation technique was adopted to measure the accuracy, where the experiment was repeated 100 times for each localization. In case of imbalanced dataset, AUC-PR is better metric than AUC-ROC as the former takes into account the information of both the classes in binary classification problem.

### Prediction with balanced dataset

The sizes of the datasets are different for different localizations (Table 1). Thus, the imbalanced dataset comes into play with the prediction using one-vs-rest strategy. For instance, in case of axon (positive) versus rest (negative), the ratio of negative to positive dataset is ~ 45:1. Use of imbalanced dataset for prediction using MLA often produces biased result towards major class^32^. There are two sampling strategies (under and over sampling) commonly used to alleviate the impact of data imbalance. In this study, we preferred SMOTE (synthetic minority over-sampling technique)^59^ technique that generates synthetic samples for the minor class. In SMOTE, synthetic observations for the minority class (class having less number of instances than the other class) are generated rather than over-sampling with replacement. The synthetic observations are introduced along the lines of the nearest neighbours of each minority class sample. Depending upon the amount of over-sampling, neighbours are randomly taken from *K*-nearest neighbours. For example, if 3 times more observations are required then only three neighbours are chosen from the *K*-nearest neighbours and one synthetic observation is generated along the direction of each. The synthetic observations are generated in 3 steps. First, the difference between the observation under consideration and its neighbour is taken. Second, the difference is multiplied with a random number between 0 and 1 and the resultant vector is added to the observation under consideration in the third step. It has been widely used in numerous bioinformatics studies in the past^60–63^.

## Results and discussion

### Analysis of kernel functions

A sample dataset with 50% sequences from each of the localization was used to choose the best fitted kernel out of 4 considered kernels, with default setting of parametric values. The prediction was made with one-vs-rest strategy. In other words, for a given localization, sequences of the remaining 7 localizations constitute the negative set. Thus, eight predictors were developed for eight different localizations. From the heat map of the AUC-ROC (Fig. 1D), it can be seen that the radial basis function (RBF) kernel yielded higher accuracy for all the eight localizations predictors across all the four different kind of feature sets. It has also been stated that RBF kernel gives best classification hyperplane due to effective training process as well as speed^39,64^. Taking the collective view, RBF kernel was utilized in the subsequent prediction analysis.

### Analysis of feature sets

With the default parameters setting of RBF kernel, prediction accuracies were further evaluated for all the four different feature sets i.e., PseDNC, DiPro, PseDNC + DiPro and PrinComp using same sample dataset as used in analyzing the kernel functions. From the ROC curves (Fig. 1E), it is seen that in most of the cases accuracies are higher for PrinComp feature set except for the localizations where the number of sequences are very less i.e., axon (16), extracellular vesicle (25) and microvesicle (21). Least accuracies are seen with DiPro features. Though PseDNC + DiPro and PrinComp have same number of features, accuracies are found to be higher for PrinComp may be due to independent nature of the principal component scores. Thus, we preferred the PrinComp features for the subsequent prediction.

### Parameter optimization analysis

Optimization of parameters is essential to obtain higher accuracy. In particular, tuning of RBF kernel width parameter (gamma: γ) and regularization parameter (cost: C) is required. Through a grid search approach, the values of the parameters were optimized. By using 50% randomly drawn sample observations for each localization (from the first dataset), optimum values of the parameters were determined. More clearly, optimum values of gamma and cost for each localization were selected out of 19 × 21 combinations of gamma and cost, where the gamma was considered as 2^–15^:2^3^ with step size 2 and cost as 2^15^: 2^–5^ with step size 2^–1^ . For all the combinations, prediction accuracies were calculated following fivefold cross validation procedure and the combination with least error was chosen as the optimum one. This process was repeated for all the eight localizations. The optimum values of parameters along with the corresponding classification error are given in Table 2. Using the optimum values of parameters, classifications were performed for all the eight localizations.

**Table 2:** Optimum parametric values of RBF kernel for prediction of miRNA in eight subcellular localizations, where sample datasets are used for optimization analysis.

Localization	γ (gamma)	C (cost)	error
Axon	0.125	2	0.05
Circulating	0.25	2	0.145
Cytoplasm	0.125	1	0.137
Exosome	0.065	8	0.121
Extracellular vesicle	0.125	2	0.11
Microvesicle	0.125	1	0.106
Mitochondrion	0.125	4	0.081
Nucleus	0.125	2	0.112

### Prediction analysis with AUC-ROC and AUC-PR

For the first dataset (positive set + ND-I), prediction was made with balanced datasets obtained after applying SMOTE (except exosome). The AUC-ROC are observed between 63–71%, whereas AUC-PR between 69–76% (Table 3). For exosome, both AUC-ROC and AUC-PR are observed to be > 97%, may be due to the large size dataset and also used without applying SMOTE. With the second dataset (positive + ND-II), it is observed that AUC-ROC are ~ 45–75% whereas the AUC-PR between ~ 50–81% (Table 3). Performance metrics are observed to be more stable for exosome and mitochondrion due to larger size datasets. On the other hand, less stable accuracies are observed for axon, extracellular vesicle and microvesicle due to smaller size datasets (Table 3). Interestingly, accuracy for exosome is less than the others in case of second dataset, may be due to that miRBase negative dataset shares a higher degree of similarity with exosome localized sequences.

**Table 3:** Prediction accuracy of the proposed model (SVM with PrinComp features).

Class	First dataset (Positive + ND-I)		Second dataset (Positive + ND-II)	
	AUC-ROC	AUC-PR	AUC-ROC	AUC-PR
Axon	0.715 (0.062)	0.761 (0.071)	0.714 (0.053)	0.765 (0.062)
Circulating	0.675 (0.037)	0.696 (0.047)	0.744 (0.027)	0.782 (0.031)
Cytoplasm	0.671 (0.033)	0.690 (0.047)	0.712 (0.027)	0.752 (0.035)
Exosome	0.971 (0.005)	0.973 (0.004)	0.452 (0.019)	0.505 (0.014)
Extracellular Vesicle	0.702 (0.058)	0.700 (0.076)	0.755 (0.043)	0.765 (0.064)
Microvesicle	0.717 (0.043)	0.792 (0.039)	0.749 (0.047)	0.810 (0.049)
Mitochondrion	0.672 (0.017)	0.734 (0.024)	0.712 (0.014)	0.773 (0.019)
Nucleus	0.635 (0.043)	0.704 (0.055)	0.646 (0.041)	0.719 (0.055)

Accuracies are measured following fivefold cross validation procedure, where the experiment was repeated 100 times.

Values inside brackets denote standard error.

### Prediction analysis with other performance metrics

Besides AUC-ROC and AUC-PR, we have also computed sensitivity, specificity, F1-score and MCC for both first (positive + ND-I) and second (positive + ND-II) datasets. Repeated fivefold cross validation technique was adopted to measure the performance metrics (similar to AUC-ROC and AUC-PR), where the experiment was repeated 100 times for each localization. The performance metrics are given in Table 4. For the first dataset, sensitivity is seen to be least for nucleus (51.3%) and highest for exosome (72.4%). Specificities are observed to be ~ 66–74%, which are higher than the sensitivities. The F1-score and MCC are found to be ~ 61–72% and ~ 52–69% respectively. Similar trend is also observed for the second dataset, where specificities (~ 68–79%) are higher than the sensitivities (~ 55–77%). Further, the F1-scores are observed between ~ 64–77%, and MCC between ~ 50–70%. Moreover, the performance metric for the second dataset are found to be higher than that of first dataset, barring few exceptions. It is also observed that the accuracies obtained with the second dataset are more stable (less standard error) than that of first dataset.

**Table 4:** Estimates of the performance metrics for the proposed model (SVM with PrinComp features).

Dataset	Localization	Sensitivity	Specificity	F1-score	MCC
First dataset (Positive + ND-I)	Axon	0.704 ± 0.027	0.740 ± 0.011	0.721 ± 0.016	0.695 ± 0.031
	Circulating	0.631 ± 0.030	0.728 ± 0.008	0.676 ± 0.018	0.613 ± 0.029
	Cytoplasm	0.657 ± 0.023	0.690 ± 0.016	0.672 ± 0.017	0.597 ± 0.033
	Exosome	0.724 ± 0.004	0.686 ± 0.004	0.706 ± 0.003	0.661 ± 0.006
	Extracellular vesicle	0.713 ± 0.021	0.731 ± 0.010	0.722 ± 0.013	0.694 ± 0.025
	Microvesicle	0.674 ± 0.027	0.742 ± 0.008	0.706 ± 0.015	0.669 ± 0.027
	Mitochondrion	0.646 ± 0.015	0.665 ± 0.011	0.654 ± 0.010	0.561 ± 0.019
	Nucleus	0.513 ± 0.048	0.741 ± 0.011	0.610 ± 0.031	0.524 ± 0.041
Second dataset (Positive + ND-II)	Axon	0.747 ± 0.023	0.791 ± 0.008	0.768 ± 0.013	0.739 ± 0.025
	Circulating	0.689 ± 0.020	0.786 ± 0.007	0.734 ± 0.012	0.679 ± 0.020
	Cytoplasm	0.717 ± 0.021	0.741 ± 0.014	0.728 ± 0.014	0.658 ± 0.026
	Exosome	0.615 ± 0.007	0.684 ± 0.006	0.644 ± 0.006	0.501 ± 0.011
	Extracellular vesicle	0.774 ± 0.013	0.787 ± 0.010	0.780 ± 0.008	0.761 ± 0.016
	Microvesicle	0.753 ± 0.021	0.787 ± 0.010	0.769 ± 0.013	0.740 ± 0.024
	Mitochondrion	0.694 ± 0.015	0.731 ± 0.011	0.711 ± 0.010	0.626 ± 0.019
	Nucleus	0.557 ± 0.035	0.788 ± 0.012	0.656 ± 0.024	0.566 ± 0.034

Accuracies are computed following fivefold cross validation procedure, where the experiment was repeated 100 times for each localization.

### Comparison with other machine learning approaches

**Figure 2:**
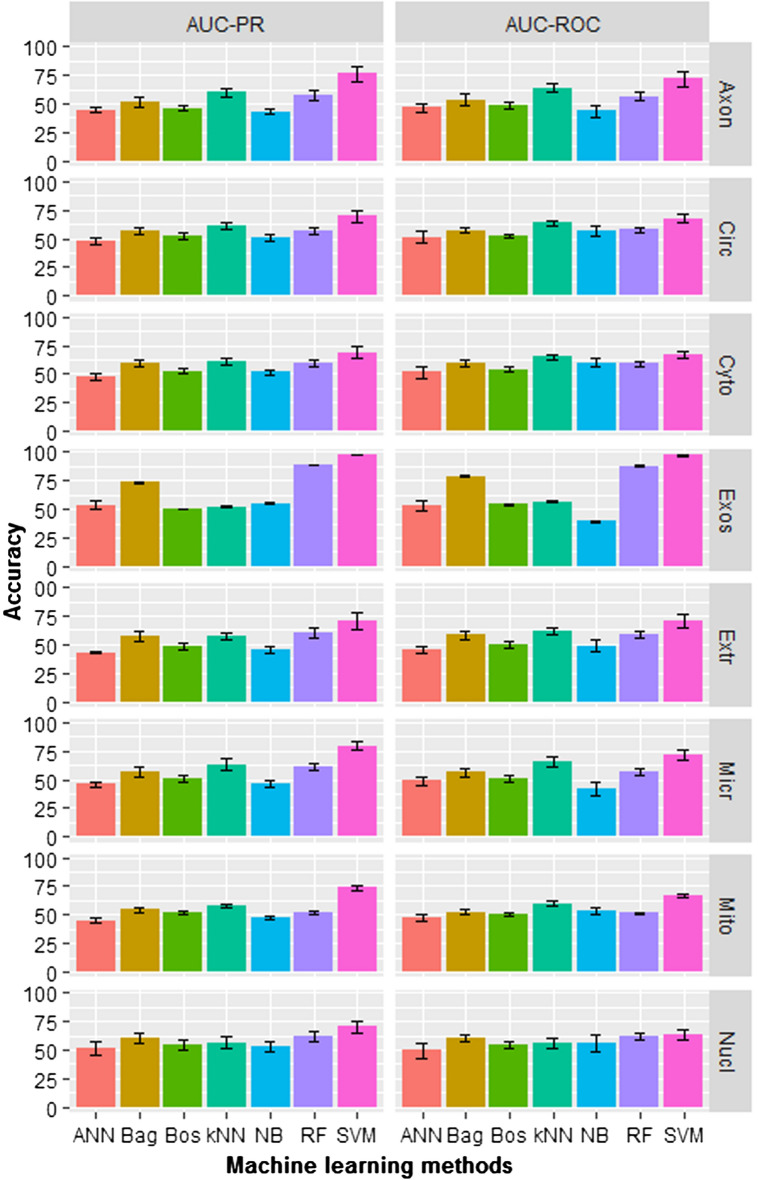
Accuracy of machine learning methods in terms of AUC-ROC and AUC-PR with regard to prediction of localizations of miRNAs.

Performance of SVM was also compared with six other well known MLAs i.e., artificial neural network (ANN)^65^, Bagging (Bag)^66^, Boosting (Bos)^67^, k-nearest neighbor (kNN)^68^, naïve Bayes (NB)^69^ and random forest (RF)^70^. Prediction performance was evaluated using the first dataset (positive set + ND-I). Different R-packages were used to implement these MLAs. List of R-packages and parameters used for execution of these techniques are provided in Supplementary Table S3. Accuracies are measured in terms of AUC-ROC and AUC-PR, where repeated fivefold cross validation technique (as mentioned in “Feature generation”) was adopted to assess the performance. Prediction accuracies are displayed in Fig. 2. It can be seen that SVM achieved highest accuracies across localization than the other classifiers, whereas ANN achieved least accuracies. Over localizations, AUC-ROC values for ANN, Bag, Bos, kNN, NB, RF and SVM are observed to be 49.09, 59.52, 51.89, 61.39, 49.81, 61.11 and 71.97 percentages, whereas AUC-PR as 47.38, 58.51, 50.66, 58.48, 49.11, 62.05 and 75.62 percentages respectively. As expected, RF performed at par or better than Bagging classifier because RF is an improved version of Bagging classifier. Interestingly, kNN is seen to be performing better than most of the classifiers across localizations, with few exceptions. Furthermore, accuracies are found to be more stable for the localizations with larger size datasets (circulating, cytoplasm, exosome and mitochondrion) and less stable with smaller size datasets (axon, extracellular-vesicle, microvesicle, nucleus). Nevertheless, SVM is found to be better than rest of the considered classifiers for predicting localizations of miRNAs.

### Prediction with independent dataset

**Figure 3:**
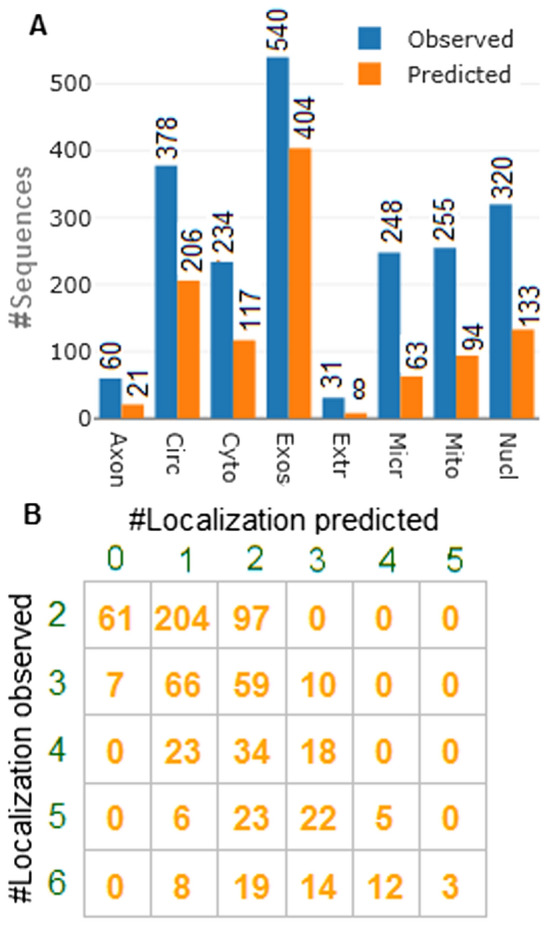
(**A**) Number of sequences observed and correctly predicted in different localizations. (**B**) Confusion matrix of the number of localizations observed and predicted.

Prediction with independent test dataset is necessary to validate newly established prediction model. Prediction was made with two models trained with two different datasets. Out of 2066 localizations (distributed over 691 sequences), 695 localisations were correctly predicted with the model trained with the second dataset (positive set + ND-II) and 1,046 were correctly predicted with the first dataset (positive set + ND-I). Though the cross validation accuracies were higher with second dataset (Table 3), less accuracies are observed with the blind dataset. Thus, it can be inferred that by using the miRBase negative dataset there is a probability of getting over prediction accuracy. On the other hand, > 50% localizations (1,046/2066) are correctly predicted with the first dataset. In particular, 35% of axon, 54.49% of circulating, 50.00% of cytoplasm, 74.81% of exosome, 25.80% of extracellular vesicle, 25.40% of microvesicle, 36.86% of mitochondrion and 41.56% of nucleus localizations were correctly predicted (Fig. 3A). Distribution of multicellular localization for the test set is shown in Fig. 3B. Out of 362 sequences that are present in exactly two localizations, both localizations are correctly predicted for 97 sequences, one localization is correctly predicted for 204 sequences, and both localizations are wrongly predicted for 61 sequences. Similarly for the 142 sequences belonging to three localizations, 1, 2 and 3 localizations are correctly predicted for 66, 59 and 10 sequences respectively, whereas all the three localizations are wrongly identified for only 7 sequences. Out of 75 sequences present in 4 localizations, 1, 2 and 3 localizations are correctly predicted for 23, 34 and 18 sequences respectively. For the sequences belonging to five localizations (56), 6, 23, 22 and 5 sequences are correctly predicted for 1, 2, 3 and 4 localizations respectively. With respect to sequences present in six localizations, 8, 19, 14, 12 and 3 are accurately predicted respectively for 1, 2, 3, 4 and 5 localizations (Fig. 3B).

### Prediction for the miRNAs of miRBase database

Prediction was also made for all the miRNA sequences (48,885 sequences) of the miRBase dataset. Less than 0.05% of sequences are predicted not to be localized in any of the considered 8 localizations. On the other hand, < 0.02% of sequences are predicted to be localized in all the 8 localizations. Besides, ~ 1.7, 8.4, 26.1, 36.3, 21.5, 5.2 and 0.7 percentages of sequences are predicted in 1, 2, 3, 4, 5, 6 and 7 localizations respectively. It is also found that 46.47, 50.21, 50.28, 59.37, 49.10, 41.97, 40.45 and 47.76 percentages of sequences are predicted into axon, circulating, cytoplasm, exosome, extracellular vesicle, microvesicle, mitochondrion and nucleus localizations respectively. For the first dataset, exosome dataset was not highly unbalanced and hence used without employing SMOTE.

### Prediction server

**Figure 4:**
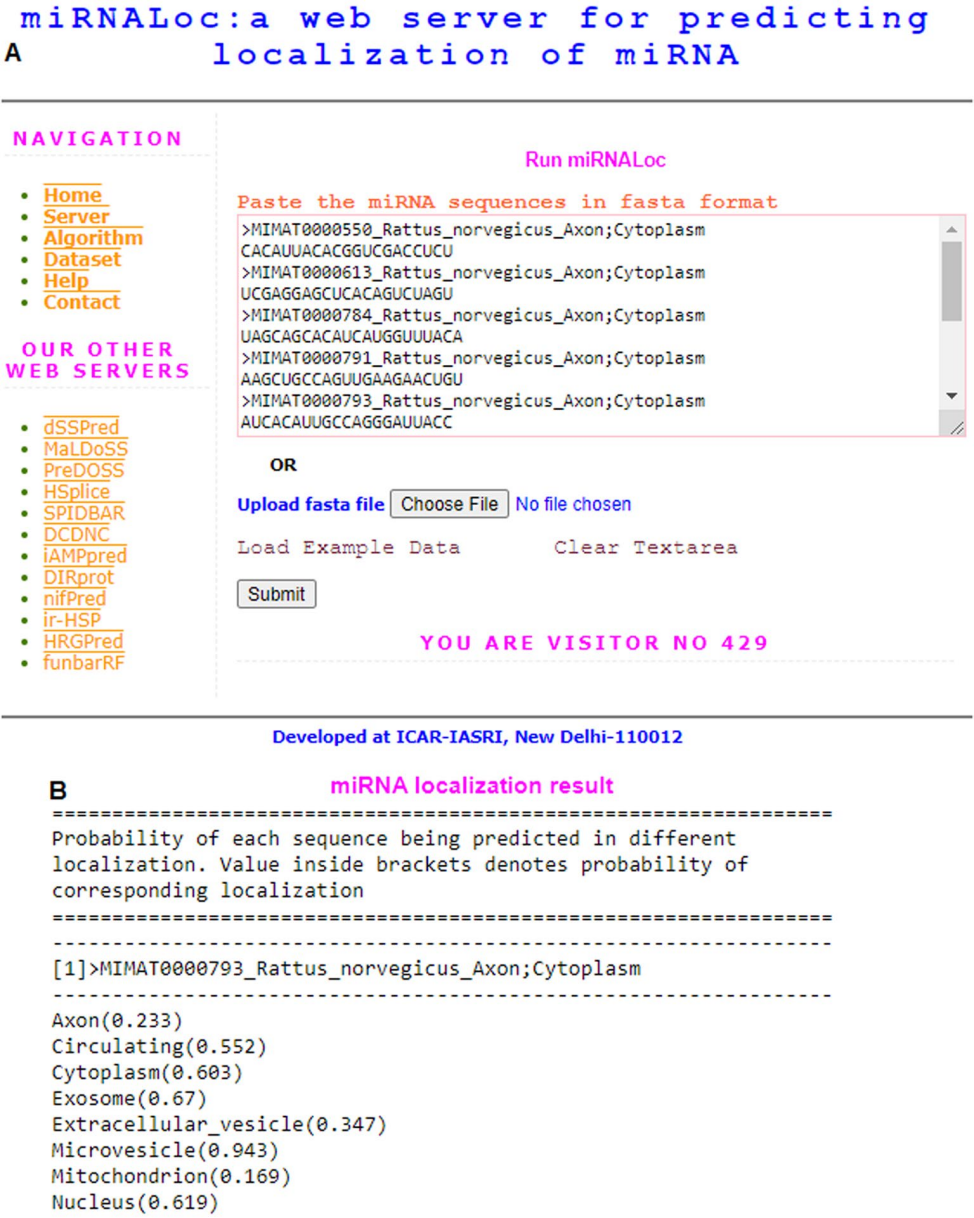
Snapshot of the (**A**) web server and (**B**) result page.

Development of web application of any computational method is essential for the users, specifically those are not familiar with the statistics or MLAs. Here, we have established a web server “miRNALoc” (https://cabgrid.res.in:8080/mirnaloc/) for predicting the localizations of miRNAs based on the proposed computational approach. The user has to supply the miRNA sequences to get the desired results. A snapshot of the server page (Fig. 4A) and resulted output for a single sequence (Fig. 4B) is shown. The result page shows the probabilities with which each sequence is predicted in eight different localizations. From the output, it is inferred that the sequence is predicted with probabilities 0.233, 0.552, 0.603, 0.67, 0.347, 0.943, 0.169 and 0.619 in localizations axon, circulating, cytoplasm, exosome, extracellular vesicle, microvesicle, mitochondrion and nucleus respectively. In other words, the sequence is predicted to be localized in circulating, cytoplasm, exosome, microvesicle and nucleus. The prediction approach is believed to supplement the localization research pertaining to other classes of ncRNA.

### Comparative analysis with existing methods

The MiRLocator and MirGOFS-based predictor are the two existing methods, as far as predicting localizations of miRNAs is concerned. Six localizations (circulating, cytoplasm, exosome, microvesicle, mitochondrion and nucleus) were considered in both the existing methods, whereas we have considered eight localizations. Further, we compared the performance of the developed model (SVM with PrinComp feature) with that of MirGOFS and MiRLocator by utilizing the same datasets that have been used in the respective models. Tenfold cross validation approach was further adopted for measuring the accuracy as has been employed in MirGOFS and MiRLocator. Same performance metrics i.e., F1-score (for MirGOFS) and average precision (for MiRLocator) were also adopted for comparison with the respective model. The developed computational model achieved F1-score of 65.77%, which is ~ 4% higher than the MirGOFS (61.2%). While compared with MiRLocator (average precision: 57.96%), ~ 4% higher of average precision is also obtained for the proposed approach (average precision: 61.82%). Thus, the established computational approach may provide higher accuracy than the considered existing methods. Moreover, none of the existing methods have been evaluated on independent dataset, whereas the developed approach correctly predicted > 50% localizations correctly while evaluated with an independent dataset. Besides, we have also developed an online prediction server for the user, whereas prediction server is not available for both the existing methods which limits their usefulness in future studies. Nevertheless, the proposed methodology is expected to supplement the available methods for predicting localizations of miRNAs.

### Advantages, disadvantages and future scope for improvement

In this study, we employed support vector machine with RBF kernel for predicting localizations of miRNAs. The SVM is seen to achieve higher accuracy than that of other models i.e., Bagging, Boosting, kNN, Naive Bayes, Random Forest and ANN algorithms. The reason may be the high generalization in prediction accuracy of SVM. Because of the imbalanced nature of the datasets, SMOTE technique was utilized to get balanced dataset and thereby higher prediction accuracy. Because of the balanced dataset obtained using SMOTE, cross validation accuracies are seen to be higher than that of accuracy achieved with the independent test dataset. Therefore, our future endeavour will be development of algorithms to get higher accuracy without balancing the different classes. Another reason of less accuracy obtained with independent dataset may be due to the use of less number of predictors (features) i.e., 33, and hence accuracy of the present methodology may be improved further by generating and including more number of discriminative features. With regard to existing localization predictors i.e., MiRLocator and MirGOFS, the developed approach may provide higher accuracy of localization prediction. Nevertheless, the present attempt is expected to add to the existing knowledge as far as computational prediction of miRNA localization is concerned.

## Conclusion

This study presents an SVM-based computational method for predicting localizations of miRNAs. Besides, a computational tool “miRNALoc” has also been established to help the biologist working in the field of RNA biology. This work is believed to supplement the biochemical methods with regard to localization study of miRNAs. The developed approach may also be useful for developing methods to predict localizations of other classes of ncRNA.

## Supplementary information

Supplementary file1

Supplementary file2

Supplementary file3

## Data Availability

All the datasets used in this study are available at https://cabgrid.res.in:8080/mirnaloc/dataset.html.
