# Publisher Correction: miRNALoc: predicting miRNA subcellular localizations based on principal component scores of physico-chemical properties and pseudo compositions of di-nucleotides

**DOI:** 10.1038/s41598-021-81881-6

**Published:** 2021-02-02

**Authors:** Prabina Kumar Meher, Subhrajit Satpathy, Atmakuri Ramakrishna Rao

**Affiliations:** ICAR-Indian Agricultural Statistics Research Institute, New Delhi, 12 India

Correction to: *Scientific Reports* 10.1038/s41598-020-71381-4, published online 03 September 2020

The original version of this Article contained a repeated error where a link to external data was incorrect.

As a result, in the Abstract,

“A user-friendly prediction server “miRNALoc” is freely accessible at https://cabgrid.res.in:8080/mirnaloc/, by which the user can predict localizations of miRNAs.”

now reads:

“A user-friendly prediction server “miRNALoc” is freely accessible at http://cabgrid.res.in:8080/mirnaloc/, by which the user can predict localizations of miRNAs.”

Additionally, in the Results and discussion section under subheading ‘Prediction server’,

“Here, we have established a web server “miRNALoc” (https://cabgrid.res.in:8080/mirnaloc/) for predicting the localizations of miRNAs based on the proposed computational approach.”

now reads:

“Here, we have established a web server “miRNALoc” (http://cabgrid.res.in:8080/mirnaloc/) for predicting the localizations of miRNAs based on the proposed computational approach.”

Finally, in the Data Availability section,

“All the datasets used in this study are available at https://cabgrid.res.in:8080/mirnaloc/dataset.html.”

now reads:

“All the datasets used in this study are available at http://cabgrid.res.in:8080/mirnaloc/dataset.html.”

These errors have now been corrected in the PDF and HTML versions of the Article.

