# Supplementary material for: miRNALoc: predicting miRNA subcellular localizations based on principal component scores of physico-chemical properties and pseudo compositions of di-nucleotides
Source: Sci Rep. 2020 Sep 3;10:14557. doi: 10.1038/s41598-020-71381-4 (PMC7471944; doi:10.1038/s41598-020-71381-4)
Supplement: Supplementary file 1 — Supplementary file1 [file 41598_2020_71381_MOESM1_ESM.docx]

miRNALoc: predicting miRNA subcellular localizations based on principal component scores of physico-chemical properties and pseudo compositions of di-nucleotides

**Prabina Kumar Meher, Subhrajit Satpathy and Atmakuri Ramakrishna Rao^*^**

ICAR-Indian Agricultural Statistics Research Institute, New Delhi-12, INDIA

*To whom correspondence should be addressed: [rao.cshl.work@gmail.com](mailto:rao.cshl.work@gmail.com)

Email

PKM: [meherprabin@yahoo.com](mailto:meherprabin@yahoo.com)

SS: [satpathyiasri@gmail.com](mailto:satpathyiasri@gmail.com)

ARR: [rao.cshl.work@gmail.com](mailto:rao.cshl.work@gmail.com)

**Supplementary Table S1.** Summary of the localization information collected from the RNALocate database.

| **Localization organelle** | **#Total Seq** | **#Uni-loc Seq** | **#Multi-loc Seq** |
| --- | --- | --- | --- |
| Axon | 85 | 18 | 67 |
| Cell body | 17 | 0 | 17 |
| Chloroplast | 2 | 2 | 0 |
| Circulating | 560 | 79 | 481 |
| Cytoplasm | 417 | 73 | 344 |
| Dendrite | 12 | 3 | 9 |
| Endoplasmic reticulum | 2 | 0 | 2 |
| Exosome | 1471 | 755 | 716 |
| Extracellular vesicle | 136 | 30 | 106 |
| Microvesicle | 362 | 22 | 340 |
| Mitochondria | 611 | 250 | 361 |
| Nucleolus | 103 | 6 | 97 |
| Nucleoplasm | 30 | 2 | 28 |
| Nucleus | 485 | 43 | 442 |
| Ribosome | 1 | 0 | 1 |
| Synapse | 49 | 9 | 40 |

#Total Seq: total number of sequences present in each localization; #Uni-loc Seq: number of sequences present in one localization only; #Multi-loc Seq: number of sequences present in more than one localization
