# Supplementary material for: miRNALoc: predicting miRNA subcellular localizations based on principal component scores of physico-chemical properties and pseudo compositions of di-nucleotides
Source: Sci Rep. 2020 Sep 3;10:14557. doi: 10.1038/s41598-020-71381-4 (PMC7471944; doi:10.1038/s41598-020-71381-4)
Supplement: Supplementary file 2 — Supplementary file2 [file 41598_2020_71381_MOESM2_ESM.docx]

miRNALoc: predicting miRNA subcellular localizations based on principal component scores of physico-chemical properties and pseudo compositions of di-nucleotides

**Prabina Kumar Meher, Subhrajit Satpathy and Atmakuri Ramakrishna Rao^*^**

ICAR-Indian Agricultural Statistics Research Institute, New Delhi-12, INDIA

*To whom correspondence should be addressed: [rao.cshl.work@gmail.com](mailto:rao.cshl.work@gmail.com)

Email

PKM: [meherprabin@yahoo.com](mailto:meherprabin@yahoo.com)

SS: [satpathyiasri@gmail.com](mailto:satpathyiasri@gmail.com)

ARR: [rao.cshl.work@gmail.com](mailto:rao.cshl.work@gmail.com)

**Supplementary Table S2.** Physico-chemical properties of di-nucleotides obtained from DiProDB database.

| **ID (DiProDB)** | **Property** | **AA** | **AC** | **AG** | **AU** | **CA** | **CC** | **CG** | **CU** | **GA** | **GC** | **GG** | **GU** | **UA** | **UC** | **UG** | **UU** |
| --- | --- | --- | --- | --- | --- | --- | --- | --- | --- | --- | --- | --- | --- | --- | --- | --- | --- |
| 24 | Shift | -0.08 | 0.23 | -0 | -0.06 | 0.11 | -0.01 | 0.3 | -0 | 0.07 | 0.07 | -0.01 | 0.23 | -0.02 | 0.07 | 0.11 | -0.1 |
| 29 | Hydrophilicity | 0.023 | 0.083 | 0.04 | 0.09 | 0.118 | 0.35 | 0.19 | 0.38 | 0.048 | 0.15 | 0.07 | 0.16 | 0.112 | 0.36 | 0.22 | 0.39 |
| 31 | Hydrophilicity | 0.04 | 0.14 | 0.08 | 0.14 | 0.21 | 0.49 | 0.35 | 0.52 | 0.1 | 0.26 | 0.17 | 0.27 | 0.21 | 0.48 | 0.34 | 0.44 |
| 101 | Slide | -1.27 | -1.43 | -1.5 | -1.36 | -1.46 | -1.78 | -1.89 | -1.5 | -1.7 | -1.4 | -1.78 | -1.43 | -1.45 | -1.7 | -1.5 | -1.3 |
| 102 | Rise | 3.18 | 3.24 | 3.3 | 3.24 | 3.09 | 3.32 | 3.3 | 3.3 | 3.38 | 3.22 | 3.32 | 3.24 | 3.26 | 3.38 | 3.09 | 3.18 |
| 103 | Tilt | -0.8 | 0.8 | 0.5 | 1.1 | 1 | 0.3 | -0.1 | 0.5 | 1.3 | 0 | 0.3 | 0.8 | -0.2 | 1.3 | 1 | -0.8 |
| 104 | Roll | 7 | 4.8 | 8.5 | 7.1 | 9.9 | 8.7 | 12.1 | 8.5 | 9.4 | 6.1 | 12.1 | 4.8 | 10.7 | 9.4 | 9.9 | 7 |
| 105 | Twist | 31 | 32 | 30 | 33 | 31 | 32 | 27 | 30 | 32 | 35 | 32 | 32 | 32 | 32 | 31 | 31 |
| 106 | Stacking energy | -13.7 | -13.8 | -14 | -15.4 | -14.4 | -11.1 | -15.6 | -14 | -14.2 | -17 | -11.1 | -13.8 | -16 | -14 | -14 | -14 |
| 110 | Enthalpy | -6.6 | -10.2 | -7.6 | -5.7 | -10.5 | -12.2 | -8 | -7.6 | -13.3 | -14 | -12.2 | -10.2 | -8.1 | -10 | -7.6 | -6.6 |
| 111 | Entropy | -18.4 | -26.2 | -19 | -15.5 | -27.8 | -29.7 | -19.4 | -19 | -35.5 | -35 | -29.7 | -26.2 | -22.6 | -26 | -19 | -18 |
| 112 | Free energy | -0.9 | -2.1 | -1.7 | -0.9 | -1.8 | -2.9 | -2 | -1.7 | -2.3 | -3.4 | -2.9 | -2.1 | -1.1 | -2.1 | -1.7 | -0.9 |
| 113 | Free energy | -0.93 | -2.24 | -2.1 | -1.1 | -2.11 | -3.26 | -2.36 | -2.1 | -2.35 | -3.4 | -3.26 | -2.24 | -1.33 | -2.4 | -2.1 | -0.9 |
| 114 | Enthalpy | -6.82 | -11.4 | -10 | -9.38 | -10.4 | -13.4 | -10.6 | -10 | -12.4 | -15 | -13.4 | -11.4 | -7.69 | -12 | -10 | -6.8 |
| 115 | Entropy | -19 | -29.5 | -27 | -26.7 | -26.9 | -32.7 | -26.7 | -27 | -32.5 | -37 | -32.7 | -29.5 | -20.5 | -33 | -27 | -19 |
