# Supplementary material for: miRNALoc: predicting miRNA subcellular localizations based on principal component scores of physico-chemical properties and pseudo compositions of di-nucleotides
Source: Sci Rep. 2020 Sep 3;10:14557. doi: 10.1038/s41598-020-71381-4 (PMC7471944; doi:10.1038/s41598-020-71381-4)
Supplement: Supplementary file 3 — Supplementary file3 [file 41598_2020_71381_MOESM3_ESM.docx]

miRNALoc: predicting miRNA subcellular localizations based on principal component scores of physico-chemical properties and pseudo compositions of di-nucleotides

**Prabina Kumar Meher, Subhrajit Satpathy and Atmakuri Ramakrishna Rao^*^**

ICAR-Indian Agricultural Statistics Research Institute, New Delhi-12, INDIA

*To whom correspondence should be addressed: [rao.cshl.work@gmail.com](mailto:rao.cshl.work@gmail.com)

Email

PKM: [meherprabin@yahoo.com](mailto:meherprabin@yahoo.com)

SS: [satpathyiasri@gmail.com](mailto:satpathyiasri@gmail.com)

ARR: [rao.cshl.work@gmail.com](mailto:rao.cshl.work@gmail.com)

**Supplementary Table S3**. List of R-packages and the functions with parameters used for executing different machine learning algorithms (MLAs).

| **MLA** | **R-package** | **Function** | **Parameter** |
| --- | --- | --- | --- |
| RF | *randomForest* | *randomForest* | ntree = 500, mtry = sqrt(P), replace = TRUE, sampsize = number of observations |
| ANN | *RSNNS* | *mlp* | size = 5, maxit = 100, initFunc = Randomize_Weights, learnFunc = Std_Backpropagation, hiddenActFunc = Act_Logistic |
| Boosting | *ada* | *ada* | loss = exponential, type = discrete, iter = 50, nu = 0.1, bag.frac = 0.5, max.iter = 20, delta = 10^(−10) |
| Bagging | *ipred* | *bagging* | nbagg = 500 |
| kNN | *class* | *knn* | k=5 |
| Naive Bayes | *klaR* | *NaiveBayes* | No parameter setting is required. |
